# A critical narrative questioning the assessment of physical literacy: foster authentic human flourishing across the life course

**DOI:** 10.3389/fspor.2024.1353386

**Published:** 2024-05-17

**Authors:** Yan Huang

**Affiliations:** Faculty of Sports and Physical Education, Xinyang Normal University, Xinyang, China

**Keywords:** physical literacy, physical literacy assessment, critical narrative, human flourishing, lifecourse

## Abstract

The study provides a summary, reflection, and clarification on the existing literature amid the recent rise in physical literacy assessment through a critical narrative that will contribute to future developments. In this review, the author addressed the significance of acknowledging the ultimate goal of assessing physical literacy throughout the life course is to foster authentic human flourishing. The study questioned the assessment practice in detail by discussing whether it is to translate and validate or to establish locally and to quantify or to specify qualitatively. The current tendency of a comprehensive dimension but not an inclusive journey was found. Subject to limitation, the merits of this study still stand and, moreover, further point to valuable areas for further inquiry that may refine and foster physical literacy and authentic human flourishing across the life course. And based on the arguments, future direction and recommendations when conducting assessment were discussed.

## Introduction

### Background

Establishing, implementing, and updating assessment methods has always been highly valued in education and public health. Traditionally, a common view links the assessment process with the screening mechanism (1). In the 21st century, scholars and experts recognized that assessment is significant in learning in and outside the classroom (2). And advanced and detailed assessments have further contributed to policy-making by accumulating comprehensive materials encompassing assessment practices from around the world (3). While in public health, measurements and evaluations are also common and have a long history (4). In many cases, health-related assessments involve the assessment of risks (5, 6), especially under the influence of the global pandemic for the past few years, many scholars have boosted their research on health risk assessments and control (7). In pursuit of positive status and health, evaluation scales of well-being have been proposed and implemented in clinical psychology as well (8, 9). In recent years, health literacy assessment tools have been experimented with in various countries and regions to promote critical knowledge and skills for the public and communities (10–14).

Why is it crucial to develop and update the assessment in education and public health?

Dating back two decades ago, Broadfoot and Black (3) stated that an “evaluation society” has become a reality in the modernization process since assessment is essential to ensure quality and equality and, in a way, to promote reform and progress. Valid and reliable measurement tools generate valuable scientific data that drives experimentation, and intervention, and facilitates effective problem-solving systems. In general, most educational issues and challenges within public health including the maintenance of bodily functions, diseases, and health care, are closely linked to the relatively opposite ends of our life course. At the start of the life journey, education lights us up and cultivates us by equipping individuals with knowledge, abilities, social skills, and insights into facing society. Entering a later stage, addressing public health challenges contributes to ensuring better survival and aging.

In addition, there are emerging social issues or public health challenges in the changing society, which require stakeholders to constantly review and update policies and methods as well as to introduce new insights respectively. Thus, reviewing and updating related assessments promptly plays a crucial role.

At present, what are some of the noteworthy changes and updates in our education and public health? The continuous revolution of electronic technology has changed the leisure lifestyle among groups of people and posed a crucial challenge of negative health outcomes for physical inactivity, especially sedentary behavior. Schools, as major educational settings around the world are responsible for the performance of students in academic subjects. But schools are more than that: they should ensure students have a positive and healthy environment to grow, and also follow a holistic developmental goal to cultivate the physical, mental, emotional, and social development of students (15). In this context, the developmental concept of “physical literacy,” which encompasses physical, cognitive, emotional, and emphasizing social domains at the same time, has been gradually introduced into the education system worldwide and has become a new indicator in educational research today. Indeed, physical literacy has been increasingly seen as an emerging public health agenda (16). The number and proportion of older people are increasing globally with the intensification of the aging process of the population regarded as one of the non-negligible social trends of the 21st century. Therefore, it is well worth it to encourage among older adults active cultivation and development of physical literacy—including physical, cognitive, and psychological health and well-being—under the promotion of active aging publicizing efforts.

### The concept

What is physical literacy, and why is it important to address the need to review, explore, and question its assessments, especially in educational and public health settings? Compared to the previous terminologies concerning embodiment either for skills acquisition or for health outcomes, what are the updates and optimizations that deserve our special attention? Early concepts, for instance physical training, fitness, physical performance, and physical capacity are normally straightforward cultivations that only involve the physical aspect while the term physical education is often confined to the school settings. Physical literacy, a holistic concept with a wider scope, consists of not only the fundamental physical domain but also considers the cognitive and affective domain of each individual throughout the whole life course. As a multidimensional construct, physical literacy has a long history in use (17) but has been welcomed and embraced by global educators, practitioners, researchers, and policymakers in recent years. Currently, the widely accepted definition of physical literacy involves motivation, confidence, physical ability, knowledge and understanding to value and assume responsibility for lifelong engagement in physical activity (18). This construct arose from Whitehead's years of exploration and inspiring philosophical debate on the underpinnings, attributes, and future directions of said concept (19). Physical literacy rejects a metaphysical dualism that separates the human body and mind when involved in physical activities and sports. Instead, it advances that monism or holistic movement guidelines underpin the construct facilitated with physical, cognitive, and affective attributes. Existentialism and phenomenology are also emphasized to allow for the active interaction with the natural and social environment (20). Based on the Whiteheadian concept and its philosophical underpinnings, different countries and regions have further built upon it according to local interpretations of the definition and construction of the framework in a way that adapts to their cultural contexts (21–23). Whether the Whiteheadian understanding, a definition now introduced by the International Physical Literacy Association as well, or its reinterpretation from diverse regional cultures, the concept of physical literacy is multi-dimensional from a horizontal dimension. This mainly includes the traditional physical domain, cognitive domain, and our affective dimension, which mainly refers to our motivation, confidence and emotions and psychological being while we participate in physical activities and interact with the people and environment around the process. From the perspective of vertical extension, physical literacy is a concept that runs through the whole life cycle, as Cairney et al. (24) pointed out physical literacy is a journey, and the journey metaphor means that it is worth noting that each person has their unique physical literacy journey due to their different physical endowments, so from the birth to the end of life, physical literacy can be a guiding and developmental program that leads a life course.

### Aims

There is a boom in conducting physical literacy-related research. However, previous empirical research mainly dealt with one or a single aspect measurement tool, while current reviews either show most interest in one population or just a summary that systematically analyzes the strengths and limits of each measure included. Therefore, the author conducted this critical narrative review in order to cover all the main aspects as well as commonly used and newly adopted measures in practice over the lifespan. The express goal is then to explore and argue for the ultimate goal of physical literacy assessment, question the prevailing tendencies, and add new insights to improve later assessment direction, design, and delivery. In short, the research questions are as follows: Were we following the right goal? Were we respecting nature and adopting the right methodology? Did we consider all essential domains and settings?

### Significance

The study addresses this issue of significance by providing a summary, reflection, and clarification on the existing literature amid the recent rise in research, which will contribute to future developments. In practice, for instance, from the perspective of education, the development of physical literacy is conducive to the overall and healthy development of students, especially motivation and cultivation of students' active participation in sports. Through more scientific and human-oriented evaluation, more research or public projects fostering physical literacy among students could be delivered smoothly and effectively. From the perspective of public health, the development of physical literacy, especially for older adults, can greatly enhance the positive effect of active aging, encourage the elderly to actively cope with various challenges, and also relieve the pressure on public medical resources. Therefore, it is worth promoting the emphasis and development of physical literacy throughout the life course and encouraging individuals to take their own physical literacy journey. Therefore, for future applications, we need more inclusive assessment tools that span a wider range including aging groups, and even adapt to groups with different chronic diseases or other health needs, so as to reach more people and promote health and well-being broadly. Additionally, it is key to promote scholars' careful assessment and evaluation of physical literacy as an important part of a developmental project or framework.

## Method

In this study, the author takes stock of the growing interest and practice in physical literacy assessment. The study examined the literature for commonly used measurement tools as well as newly adopted ones, e.g., data derived from databases ranging from SPORTDiscus, PubMed, Scopus, ScienceDirect, EBSCO, ERIC, and Web of Science. These databases cover substantial research concerning education, public health, and physical activity-oriented articles. The inclusion criteria were: (1) peer-reviewed studies; (2) articles in the English language. The exclusion criteria were also adopted: (1) studies not applying a validated measurement tool; (2) reports, editorials, and newsletters. Therefore, English-language, peer-reviewed published articles containing measurement of physical literacy have been investigated. A Boolean logic combinations search strategy has been also adopted within the electronic databases, including “physical literacy assessment”. Inverted commas were applied to the term “physical literacy assessment” to ensure searches would find studies concerning physical competence as opposed to searches related to “physical literacy” and “assessment”. Keywords with related constructs and understanding have been also included in this search (e.g., “physical literacy measurement” or “physical literacy evaluation”). And journal filter boxes have been marked on all searches to ensure only these publications would appear in the listing.

Further, the author investigated studies conducted from the past two decades to ascertain just how the concept is gradually adopted by academics. The method has evolved from a “screening/synthesizing/summarizing” procedure to a philosophical one premised on “debating/arguing/critically narrating.” At the early stage, the author screened and selected articles based on keywords and abstracts, and then according to the articles included, synthesizing the assessments and summarizing key contents to be discussed was conducted. Then, during the main stage, the author drew the main lines of argumentation. At the final stage, the critical narrative was completed. Below, the arguments will be stated in the following discussion and the study will address the conclusion and implications for future implementation as well.

## Discussion

This narrative firstly demonstrated that prior to any design and delivery, the ultimate goal of assessing physical literacy throughout the life course shall be acknowledged, which is to foster authentic human flourishing. And the author questioned the tendency and the assessment in detail in the following section.

### Fostering authentic human flourishing

After synthesizing the main findings and overall goals in the literature, the author found that human flourishing is a desirable outcome that should be addressed properly with regard to the cultivation of physical literacy. Previous studies explored the relationships between human flourishing and physical literacy and further pointed out that the process of fostering physical literacy can contribute to human flourishing (25). Earlier, in the view of Rasmusssen (26), human flourishing is a term concerning human nature and ethics that originates in ancient Greek terminology that stands for a state of being happy. In recent years it has been introduced into positive psychology as a new theory (27). Human flourishing does not arise only in Western culture: in the Eastern context, flourishing is clearly found in Buddhism (28), where it is interpreted as a state of healthy balance of mood and mind. In short, human flourishing and physical literacy actually share a similar diverse cultural basis. When delved in deeply, based on Rasmussen (26), Durden-Myers and scholars (29) expand on the characteristics of human flourishing and its attributes, i.e., objective good, inclusivity, individualism, agent-dependency, self-directedness, and being socially constructed. These attributes echo the aforementioned nature of physical literacy in respective domains and across the life course of each individual's own physical literacy journey. Physical literacy proposes a conceptual model for education and lays out the agenda for public health research. Moreover, human flourishing has been proposed not only in the field of positive psychology but also endorsed by medical studies and population health frameworks (28). Beforehand, in the discipline of education, human flourishing has been proposed as the goal for students along three axes: (1) in the cognitive domain, to help with the development of knowledge and wisdom, (2) in the physical domain, to encourage positive behavior and a good state of health, and (3) in the affective domain, to foster better decision-making and social enjoyment (30). These comprehensive aspects justify the multi-dimensional facets of physical literacy. Before critically assessing physical literacy, the author clarifies and emphasizes that the ultimate goal of engaging physical literacy is to foster authentic human flourishing across the life course. With human flourishing as the foundation, the study proceeds to review and discuss the current assessment practice, and the arguments are as follows.

### To translate and validate or to establish locally?

A great number of assessment research focuses on the translation and validation of well-established measurement tools from one culture into another. Now, can we really make it a perfect fit and achieve the ultimate goal of human flourishing through direct “translation-validation”? Following are two cases as examples that raise some concerns.

Over the years, Canada has been the pioneer in designing and programming inclusive assessment tools. Among these, there is The Canadian Assessment of Physical Literacy (CAPL) (31), which has proven to have a high level of validity among children aged 8–12 (32) to assess daily behavior, motivation and confidence, physical competence, knowledge and understanding of participants. This assessment tool has been included in many Canadian educational programs and evidence-based research. This Canadian assessment tool has been translated to and validated in Greece (33) and Hong Kong (34) where it has been included in local programs. Similarly. The perceived Physical Literacy Instrument (PPLI) (35, 36), which was an instrument established in Hong Kong, has been recently translated and adopted in Spain (37) and France (38).

The question arises, however, whether it is appropriate to “borrow” the assessment from another cultural setting even when translation and adoption efforts are implemented? This study contends that it would be more significant if the assessment had a fully indigenous or local cultural underpinning. Different regions have different political and economic systems, and aspects of social and cultural activities are diverse accordingly, not to mention the critical issues among education and public health. Although the values of universal education and the development of students appeal to a global consensus, there are differences in practice and distinctions shown in the process of educational development or reforms in each region, such that corresponding differences in the cognition and views among students in these regions cannot be merely equated or translated without significant modification. Such challenges cannot be solved by simply “translating” a framework even when putatively trying to adjust it to local cultural regulations. Just as different primary schools in the same region, for instance, may not share the same teaching plans or course outline, all things being equal, taking a much more complex physical literacy instrument that has been developed based on and to fit a specific population and its inherently unique needs is beyond the scope of mere “translation-validation.” This difference is even more obvious within public health. Different regions have different levels of tension on public resources, and each has its complex system for responding to public health issues and social care policies; thus the health-related cognition, degree of physical activity engagement, and lifestyle will not share the same standard. From the perspective of anthropology, those aforementioned areas are diverse ethnically, and in terms of physiological characteristics, and the aging process. Accordingly, the same set of measurements, interpretation, and classification obviously cannot perfectly be matched to or refer to the same population (especially when further comparisons are warranted).

Human flourishing must be culturally sensitive. When introducing the concept of physical literacy, the author mentioned that there have been countries and areas that actively make an effort to fit physical literacy—from concept to framework—to the local culture. In this light, the study found the positive case of a physical literacy instrument developed in Australia (21, 39) which, aside from having spent years becoming familiar and practicing with the Canadian model, was specifically designed, from the concept construction to the assessment guidelines exploration, to fit the local culture.

Therefore, concerning the issue of translating-validating or establishing an assessment guideline, tool, or system for practice, this study contends that local culture shall be respected and drive research, guidelines, and implementation processes from the ground up and more merely as “translated adaptation.” It is appropriate to conduct further validation within the same culture system for stronger evidence or larger significance. When a distinction between the cultural background and the original instrument of the framework is found, the author recommends starting with the construction of the basic concept of physical literacy with the local context in mind and, based on this underpinning, establishing the assessment of physical literacy in a culturally sensitive manner. Besides the aforementioned case of Australia, The Greater China Region, which was significantly different from the western in behavior, culture and other forms, has also made a positive move in showing the cultural adaptation of physical literacy in Chinese context through experts planning and mapping, to appraisal, synthesis and recommendation phases to explore the concept, meaning and key elements of physical literacy in Chinese culture and society (22). It is a recommended start especially when the distinction exists and with the adapted key elements raised, more focused and detailed assessment methods could be applied to the practice.

### To quantify or to specify qualitatively?

It is not surprising that most assessment practices show interest in accumulating quantitative data or objectively conducting the assessment. It meets the tradition of the physical tests. In terms of the affective and cognitive domains of physical literacy, previous research has also preferred adopting self-reported questionnaires or Likert scales. Nonetheless, for a purportedly holistic framework as that of physical literacy, this begs the question of whether the qualitative aspect is missing. Would the assessments not benefit from more qualitative and self-directed specifications?

Indeed, there is little qualitative research in the physical literacy assessment practices. Moreover, among those few, some are just supplementary and others merely rely on previous research on the experience or the perceptions of the physical literacy program (40, 41). In 2022, Korean educators and scholars proposed a new qualitative approach that combines E-Portfolios and interviews in the charting of physical literacy journeys among students. The features of the e-portfolio action were categorized as (a) digital fumblers, (b) superficial participants, (c) passionate creators, and (d) journey reflectors (42). This new attempt gives new insight as to how to conduct specific in-depth assessment that also takes into account quality.

Self-directedness is one of the attributes of human flourishing: humans flourish only if they experience (30). Congruently, physical literacy also requires an individual to take responsibility to engage in physical activities for life. In this regard, there is still more to be explored in the qualitative approach as it needs to take into account each specific and self-centered physical literacy journey within diverse groups and populations. To date, based on a large number of quantitative assessments, a lot of data and empirical results have been acquired but there is a clear need for assessments that take into account the humanistic facet of physical literacy such that they develop more qualitative, specific, and personalized assessment systems especially customized for different groups. Mixed methods are also recommended in the assessment process. The author has just finished one such attempt that combined qualitative exploration through life history interviews, complemented by quantitative measures including Short Physical Performance Battery and Perceived Physical Literacy Instrument to explore the physical literacy journeys among older adults in Hong Kong comprehensively and deeply (43). In future practice, it is suggested to integrate quantitative methods and qualitative ones in large-scale projects especially when they are empirical studies within specific settings.

### A comprehensive dimension but not an inclusive journey

Some of the earlier attempts at physical literacy assessments show a preference for the fundamental physical domain. In this regard, there are two Canadian tools that were introduced in earlier practice: the Physical Literacy Observatory Tool (PLOT) (44) was designed to assess the fundamental movement skills of children aged 6 months to 6 years, the Physical Literacy Assessment for Youth (PLAY) focused on the measurement of the physical domain among children, especially young athletes aged 7–12 years (32). Similarly, in the UK, fundamental movement skills were also a concern for the Youth Sport Trust (45), which designed an application for the use of physical educators to evaluate skills. Although it has been further updated it to Skills2Achieve, the affective and cognitive domains of physical literacy are still being neglected.

The holistic nature of the concept requires a comprehensive assessment. As an example of how this can be implemented, The Society of Health and Physical Educators (SHAPE) America (46) developed outcome standards, including psychomotor, cognitive, and affective domains for K-12 physical education in the US. It showed a relatively comprehensive combination in the earlier years of physical literacy assessment construction. The aforementioned PPLI records the knowledge and understanding, self-expression and communication, sense of self, and self-confidence of physical literacy. Consistent with my position, this standard is developed in a customized and culturally sensitive way. Recent common assessment practices show a balanced approach that includes all the essential defining aspects of physical literacy. In this manner, CAPL now takes all daily behavior, motivation and confidence, physical competence, knowledge and understanding into account as the outcome measure items. The aforementioned Australian guidelines also consider all the aspects based on their local context and concept.

The refinement in the comprehensiveness of the assessment marks significant progress. However, this still does not seem to be balanced duly enough if we consider the application of physical literacy across the life course. The assessment tools abovementioned are mainly suitable for adolescents or developed within the field of school education, and some even have strict age restrictions (e.g., PLOT: 6 months-6 years old; CAPL: 8–12 years old). At present, there is a lack of design and practice of physical literacy assessment for adults, especially older adults, with regard to the public health agenda. As the study has already stated, a holistic measurement system needs to include every aspect of physical literacy but, crucially, also take every stage of life into account (16). Hence, the study presently makes the case for further research on the later stages of the physical literacy journey.

## Conclusion

This study was inspired by the growing interest in physical literacy assessment. While there are limiting factors—only articles in English were examined, interpretation was based on the authors’ personal research experience and understanding within the field, and no further result analysis with similar assessment tools was conducted—the merits of this study still stand and, moreover, further point to valuable areas for further inquiry that may refine and foster physical literacy and authentic human flourishing across the life course.

Having reviewed commonly used measurement tools and newly adopted ones, the author has posited that human flourishing is the ultimate goal when conducting physical literacy assessments. Further, the study discussed how current research shows a preference for translating and validating well-established tools while designing the assessment based on local cultural contexts has been neglected. There is a tendency to quantify physical literacy and the domains included, especially within educational settings. In this regard, this study recommends establishing the assessment of physical literacy carefully to fit the local culture (not just translated and validated). It is suggested that linking the concept of and elements within physical literacy to the local history, behavior and practice will contribute to more focused and detailed assessment methods, which will be the foundation of an effective project. Moreover, the spirit and principle are to encourage a more humanistic consideration with more qualitative, specific, and personalized assessment systems especially customized for different groups. Therefore, more specific qualitative approaches are encouraged and could be applied in a project together with quantitative ones as a mixed design. Finally, there is a need to encourage older adults to be more engaged in physical activities both physically and mentally and increase their awareness of the individual's body and health through focusing also on the later stages of the physical literacy journey, which can greatly enhance the active aging effectiveness. In the future implementation, more inclusive assessment tools that span a wider range including aging groups, and even adapt to groups with different chronic diseases or other health needs are encouraged to take each individual into account vis à vis the public health agenda.

## References

[1] Torrance H. The role of assessment in educational reform. In: Torrance H, editors. Evaluating Authentic Assessment: Problems and Possibilities in new Approaches to Assessment. Buckingham: Open University Press (1995). p. 144–56.

[2] Buhagiar MA. Classroom assessment within the alternative assessment paradigm: revisiting the territory. Curric J. (2007) 18(1):39–56. 10.1080/09585170701292174

[3] Broadfoot P Black P. Redefining assessment? The first ten years of assessment in education. Assess Educ. (2004) 11(1):7–26. 10.1080/0969594042000208976

[4] Ware J. The status of health assessment 1994. Annu Rev Public Health. (1995) 16(1):327–54. 10.1146/annurev.pu.16.050195.001551 7639876

[5] Buchanan RL Whiting RC. Risk assessment: a means for linking HACCP plans and public health. J Food Prot. (1998) 61(11):1531–4. 10.4315/0362-028X-61.11.1531 9829200

[6] Levy JI Buonocore JJ Von Stackelberg K. Evaluation of the public health impacts of traffic congestion: a health risk assessment. Environ Health. (2010) 9:1–12. 10.1186/1476-069X-9-1 20979626 PMC2987789

[7] Jost TS. Considering race and ethnicity in COVID risk assessments — legal concerns and possible solutions. N Engl J Med. (2022) 387(6):481–3. 10.1056/NEJMp2206524 35929820

[8] Holland J Murphy D Joseph S. Assessment of well-being in the clinic: using the state version of the short scale of general well-being as a clinical outcome measure. J Clin Psychol. (2021) 77(7):1629–43. 10.1002/jclp.23166 34096060

[9] Kobau R Sniezek J Zack MM Lucas RE Burns A. Well-being assessment: an evaluation of well-being scales for public health and population estimates of well-being among US adults. Appl Psychol. (2010) 2(3):272–97. 10.1111/j.1758-0854.2010.01035.x

[10] Guzys D Kenny A Dickson-Swift V Threlkeld G. A critical review of population health literacy assessment. BMC Public Health. (2015) 15:215. 10.1186/s12889-015-1551-6 25885742 PMC4351936

[11] Jensen NH Aaby A Ryom K Maindal HT. A CHAT about health literacy—a qualitative feasibility study of the conversational health literacy assessment tool (CHAT) in a Danish municipal healthcare centre. Scand J Caring Sci. (2021) 35(4):1250–8. 10.1111/scs.12943 33314213 PMC9290499

[12] Mansfield ED Wahba R Gillis DE Weiss BD L’Abbé M. Canadian Adaptation of the newest vital sign©, a health literacy assessment tool. Public Health Nutr. (2018) 21(11):2038–45. 10.1017/S1368980018000253 29692290 PMC10260894

[13] Platter H Kaplow K Baur C. The value of community health literacy assessments: health literacy in Maryland. Public Health Rep. (2022) 137(3):471–8. 10.1177/00333549211002767 33706612 PMC9109546

[14] Senahad N Loahasiriwong W Boonsuk S. Development of health literacy assessment tool for 9–10 years old children in Thailand. Public Health Pract (Oxford, England). (2023) 6:100420. 10.1016/j.puhip.2023.100420 PMC10506043 37727706

[15] OECD Future of education 2030. Making Physical Education Dynamic and Inclusive for 2030 International Curriculum Analysis. (2019).

[16] Huang Y Sum KWR Yang YJ Yeung NCY. Measurements of older adults’ physical competence under the concept of physical literacy: a scoping review. Int J Environ Res Public Health. (2020) 17(18):1–18. 10.3390/ijerph17186570 PMC7558186 32916990

[17] Cairney J Dudley D Kwan M Bulten R Kriellaars D. Physical literacy, physical activity and health: toward an evidence-informed conceptual model. Sports Med. (2019a) 49(3):371–83. 10.1007/s40279-019-01063-3 30747375

[18] Whitehead M. Definition of physical literacy. In: Whitehead M, editors. Physical Literacy Across the World. London: Routledge (2019). p. 8–18.

[19] Whitehead M. The concept of physical literacy. Eur J Phys Educ. (2001) 6(2):127–38. 10.1080/1740898010060205

[20] Whitehead M. Physical competence and interaction with the environment. In: Whitehead M, editors. Physical Literacy : Throughout the Lifecourse. London: Routledge (2010). p. 44–55.

[21] Keegan RJ Barnett LM Dudley DA Telford RD Lubans DR Bryant AS Defining physical literacy for application in Australia: a modified delphi method. J Teach Phys Educ. (2019) 38(2):105–18. 10.1123/jtpe.2018-0264

[22] Li MH Whitehead M Green N Ren H Cheng C-F Lin LL-C Operationally defining physical literacy in Chinese culture: results of a meta-narrative synthesis and the panel’s recommendations. J Exerc Sci Fitness. (2022) 20(3):236–48. 10.1016/j.jesf.2022.04.003 PMC9117885 35646130

[23] Tremblay MS Costas-Bradstreet C Barnes JD Bartlett B Dampier D Lalonde C Canada’s physical literacy consensus statement: process and outcome. BMC Public Health. (2018) 18(S2):1–18. 10.1186/s12889-018-5903-x PMC6167775 30285701

[24] Cairney J Kiez T Roetert EP Kriellaars D. A 20th-century narrative on the origins of the physical literacy construct. J Teach Phys Educ. (2019b) 38(2):79–83. 10.1123/jtpe.2018-0072

[25] Robinson DB Durden-Myers EJ Berg S. Physical Literacy and Flourishing (Within) Canadian School Communities. Perspectives on Flourishing Schools. Lanham, MD: Lexington Books (2018). p. 199.

[26] Rasmussen DB. Human flourishing and the appeal to human nature. Soc Philos Policy. (1999) 16(1):1–43. 10.1017/S0265052500002235

[27] Pennington JT. The Sermon on the Mount and Human Flourishing: A Theological Commentary. Ada, Michigan, US: Baker Publishing Group (2017).

[28] Levin J. Human flourishing and population health: meaning, measurement, and implications. Perspect Biol Med. (2020) 63(3):401–19. 10.1353/pbm.2020.0029 33416615

[29] Durden-Myers EJ Whitehead ME Pot N. Physical literacy and human flourishing. J Teach Phys Educ. (2018) 37(3):308–11. 10.1123/jtpe.2018-0132

[30] De Ruyter DJ. Pottering in the garden? On human flourishing and education. Br J Educ Stud. (2004) 52(4):377–89. 10.1111/j.1467-8527.2004.00274.x

[31] Healthy Active Living and Obesity Research Group (HALO). Canadian Assessment of Physical Literacy. Manual for Test Administration. Ottawa, ON, Canada: HALO (2014).

[32] Robinson DB Randall L. Marking physical literacy or missing the mark on physical literacy? A conceptual critique of Canada’s physical literacy assessment instruments. Meas Phys Educ Exerc Sci. (2017) 21(1):40–55. 10.1080/1091367X.2016.1249793

[33] Dania A Kaioglou V Venetsanou F. Validation of the Canadian assessment of physical literacy for Greek children: understanding assessment in response to culture and pedagogy. Eur Phy Educ Rev. (2020) 26(4):903–19. 10.1177/1356336X20904079

[34] Li MH Sum RKW Tremblay M Sit CHP Ha ASC Wong SHS. Cross-validation of the Canadian assessment of physical literacy second edition (CAPL-2): the case of a Chinese population. J Sports Sci. (2020) 38(24):2850–7. 10.1080/02640414.2020.1803016 32787646

[35] Sum RKW Ha ASC Cheng CF Chung PK Yiu KTC Kuo CC Construction and validation of a perceived physical literacy instrument for physical education teachers. PLoS One. (2016) 11(5):e0155610. 10.1371/journal.pone.0155610 27195664 PMC4873233

[36] Sum RKW Cheng C Wallhead T Kuo C Wang F Choi S. Perceived physical literacy instrument for adolescents: a further validation of PPLI. J Exerc Sci Fitness. (2018) 16(1):26–31. 10.1016/j.jesf.2018.03.002 PMC6323161 30662489

[37] Mendoza-Muñoz M Carlos-Vivas J Castillo-Paredes A Sum RKW Rojo-Ramos J Pastor-Cisneros R. Translation, cultural adaptation and validation of perceived physical literacy instrument-spanish version (PPLI-Sp) for adults. J Sports Sci Med. (2023) 22(3):455–64. 10.52082/jssm.2023.455 37711722 PMC10499136

[38] Gandrieau J Dieu O Potdevin F Derigny T Schnitzler C. Measuring physical literacy for an evidence-based approach: validation of the French perceived physical literacy instrument for emerging adults. J Exerc Sci Fit. (2023) 21(3):295–303. 10.1016/j.jesf.2023.06.001 37520158 PMC10373918

[39] Barnett LM Dudley DA Telford RD Lubans DR Bryant AS Roberts WM Guidelines for the selection of physical literacy measures in physical education in Australia. J Teach Phys Educ. (2019) 38(2):119–25. 10.1123/jtpe.2018-0219

[40] Bartle G. An investigation of how physical literacy is enacted in primary physical education. Qualitative Research Journal. (2023) 23(3):325–37. 10.1108/QRJ-08-2022-0117

[41] Ha AS Zeng T He Q Chan CHS Fung Y Ng JYY. Acceptability of a parent-focused program for physical literacy development among parents and children during the COVID-19 pandemic: a qualitative study. Front Public Health. (2022) 10:924434. 10.3389/fpubh.2022.924434 36187639 PMC9520592

[42] Woo P Lee O. Utilization of E-portfolios for charting the physical literacy journeys of students in secondary PE classes. IJASS(Inte J Appl Sports Sci). (2022) 34(2):95–108. 10.24985/ijass.2022.34.2.95

[43] Huang Y. Exploring and Understanding Physical Literacy Journeys of day Care Centers’ Older Adults in Hong Kong. Hong Kong: The Chinese University of Hong Kong (2023).

[44] Early Years Physical Literacy Research Team. Physical Literacy Observatory Tool (PLOT). (2017). Available online: Available online at: https://docs.wixstatic.com/ugd/05c80a_4d5d31e1f84440dab941060ff054b07e.pdf (Accessed on 1 May 2020).

[45] Youth Sport Trust. Start to Move: The ABCs of Physical Education for Early Years and Key Stage 1. (2017) Retrieved from Available online at: https://www.youthsporttrust.org/start-move (Accessed February 28, 2020).

[46] SHAPE America. National Standards & Grade-Level Outcomes for K-12 Physical Education. Champaign, IL: Human Kinetics (2014).

